# Multidisciplinary approach with stereotactic reirradiation to oligoprogressive spinal metastases from dedifferentiated osteosarcoma: a case report

**DOI:** 10.3389/fonc.2026.1929146

**Published:** 2026-10-26

**Authors:** Arkadiusz Moskwa, Paulina Chmiel, Katarzyna Kozak, Michał Wągrodzki, Piotr Rutkowski, Mateusz Spałek

**Affiliations:** 1 Department of Soft Tissue/Bone Sarcoma and Melanoma, Maria Sklodowska-Curie National Research Institute of Oncology, Warsaw, Poland; 2 Department of Pathology, Maria Sklodowska-Curie National Research Institute of Oncology, Warsaw, Poland; 3 Department of Radiotherapy I, Maria Sklodowska-Curie National Research Institute of Oncology, Warsaw, Poland

**Keywords:** case report, multidisciplinary care, oligoprogression, osteosarcoma, reirradiation, spinal cord sparing, spinal metastases, stereotactic body radiotherapy

## Abstract

**Background:**

Stereotactic body radiotherapy (SBRT) has emerged as a promising therapeutic approach for vertebral metastases, achieving high local control and safety. However, evidence on spinal reirradiation with SBRT is limited, and data are especially scarce for radioresistant histologies such as osteosarcoma.

**Case report:**

We present the first case of a second reirradiation in radioresistant metastatic osteosarcoma, with each consecutive course of radiotherapy administered at an interval of at least 6 months. After multiple surgical interventions in the cervical and thoracic spine and three lines of chemotherapy, the patient demonstrated local progression in the C-Th region of the spine, which was successfully treated with a third SBRT, achieving a partial response without any acute adverse events.

**Conclusions:**

SBRT for spinal metastases reirradiation has seen a growing trend in recent years. Preliminary studies, as well as the following case report, demonstrate its usefulness, effectiveness, and safety in a carefully selected group of patients. In this particular instance, even a third SBRT course has been demonstrated to yield clinical benefits and can be executed safely, provided that it is administered in accordance with proper technical regimens and critical organ sparing strategies.

## 1 Introduction

Bone metastases occur commonly in advanced solid tumors, constituting the third most frequent metastatic site after the lungs and liver (1, 2). One of the most frequent locations for metastases is the spine. Research indicates that up to 15% of metastases can locate in the spine, causing pathologic vertebral compression fractures and metastatic epidural spinal cord compression (3). These conditions significantly reduce the quality of life of cancer patients and result in chronic pain. Osteosarcoma is a rare, highly aggressive bone tumor that can initially originate in the spine and, in approximately 5% of cases, metastasize to the spine (4–6). Conventional external beam radiation therapy (EBRT) was previously employed as a treatment for inoperable spinal metastases, including those that had progressed following prior irradiation (7). However, randomized trials have demonstrated that EBRT exhibits moderate efficacy in both local control (LC) and pain management. Chow et al. demonstrated that re-irradiation with two distinct EBRT schedules for painful and previously irradiated bone metastases resulted in overall pain response rates of 28-32% and complete response rates of 7-8% (8). However, with the advent of advanced radiotherapy (RT) techniques, stereotactic body radiotherapy (SBRT) emerged as a significant development. This technique enables the delivery of highly conformal and ablative dose distributions with millimeter precision to body targets, thereby achieving markedly superior outcomes in the treatment of spinal metastases (9). A growing body of research has compared EBRT with SBRT for addressing painful spinal metastases. These studies have consistently shown that SBRT is more efficacious, leading to superior local control (LC) and decreased re-irradiation needs (10–12). Nevertheless, a subset of patients continues to require additional irradiation, and the existing literature on re-irradiation employing SBRT is limited. Moreover, there are no randomized clinical trials on re-irradiation of spinal metastases. A recently published study employing SBRT reirradiation for bone metastases demonstrated a local progression-free rate of 89.9% at 2 years, along with an overall survival (OS) rate of 52% at 2 years. However, acute toxicities were observed in nearly half of the patients (13).

We experienced a case of a 42-year-old woman receiving a third irradiation for a vertebral metastasis secondary to an advanced osteosarcoma. Here, we describe our case with a review of literatures. The current case report follows the CARE guidelines as provided in **Supplementary File 1**.

## 2 Case report

The following report details the case of a patient, born in 1984, who received initial treatment at an external facility for a locally malignant desmoid-type (spindle-cell) tumor of the left femur. The patient exhibited no other relevant medical history.

In February 2015, the patient underwent a resection of the distal segment of the left femur with cement filling due to a suspicious lesion within the lateral condyle of the left femur, outside of our referral center. After this procedure an MRI scan in April 2016 revealed a suspected local recurrence, prompting referral to our center for further evaluation. A subsequent review of the histopathology report from the initial procedure resulted in a modification of the diagnosis to low-grade central osteosarcoma. Then the patient continued treatment at her regional medical facility as recommended, where she received chemotherapy according to the AP3 regimen (doxorubicin at 20 mg/m² and cisplatin at 30 mg/m²) before surgical resection of the recurrence. Due to local progression, only two treatment courses were administered, followed by resection with a distal femoral endoprosthesis in October 2016. Postoperatively, adjuvant treatment consisted of one additional course of AP3 and two courses of HD-IFO (ifosfamide 3g/m²) were administered. The patient was subsequently observed. A Positron Emission Tomography - Computed Tomography (PET-CT) scan performed in 2018 revealed a metabolically active lesion within the Th2 vertebra. Consequently, in January 2019, the patient underwent an excisional biopsy at her local medical facility. A follow-upconsultation on the histopathological slides of the excised lesion and comparison with the initial femoral specimen, led to the diagnosis of high-grade dedifferentiated osteosarcoma and confirmed the diagnosis. In light of the final diagnosis, the clinic’s multidisciplinary board determined that further investigation was necessary to preclude the possibility of disease dissemination. Following the confirmation of no other metastatic lesions through computed tomography (CT) and magnetic resonance imaging (MRI) scans, the decision was made to proceed with adjuvant radiotherapy to the region of the resected spinal metastasis bed in the spine. The histopathological diagnosis and the observation of unclear resection margins guided the approach that was implemented. In July 2019 adjuvant SBRT was delivered with volumetric modulated arc therapy (VMAT), cone-beam computed tomography (CBCT) image guidance, and a 6 MV photon beam. The target was the post-resection bed at T2, defined on CT-PET and CT-MRI fusion (**Figure 1**), with adequate margin of healthy tissues. A total dose of 27 Gy in 3 fractions was prescribed to the 77% isodose line.

**Figure 1:**
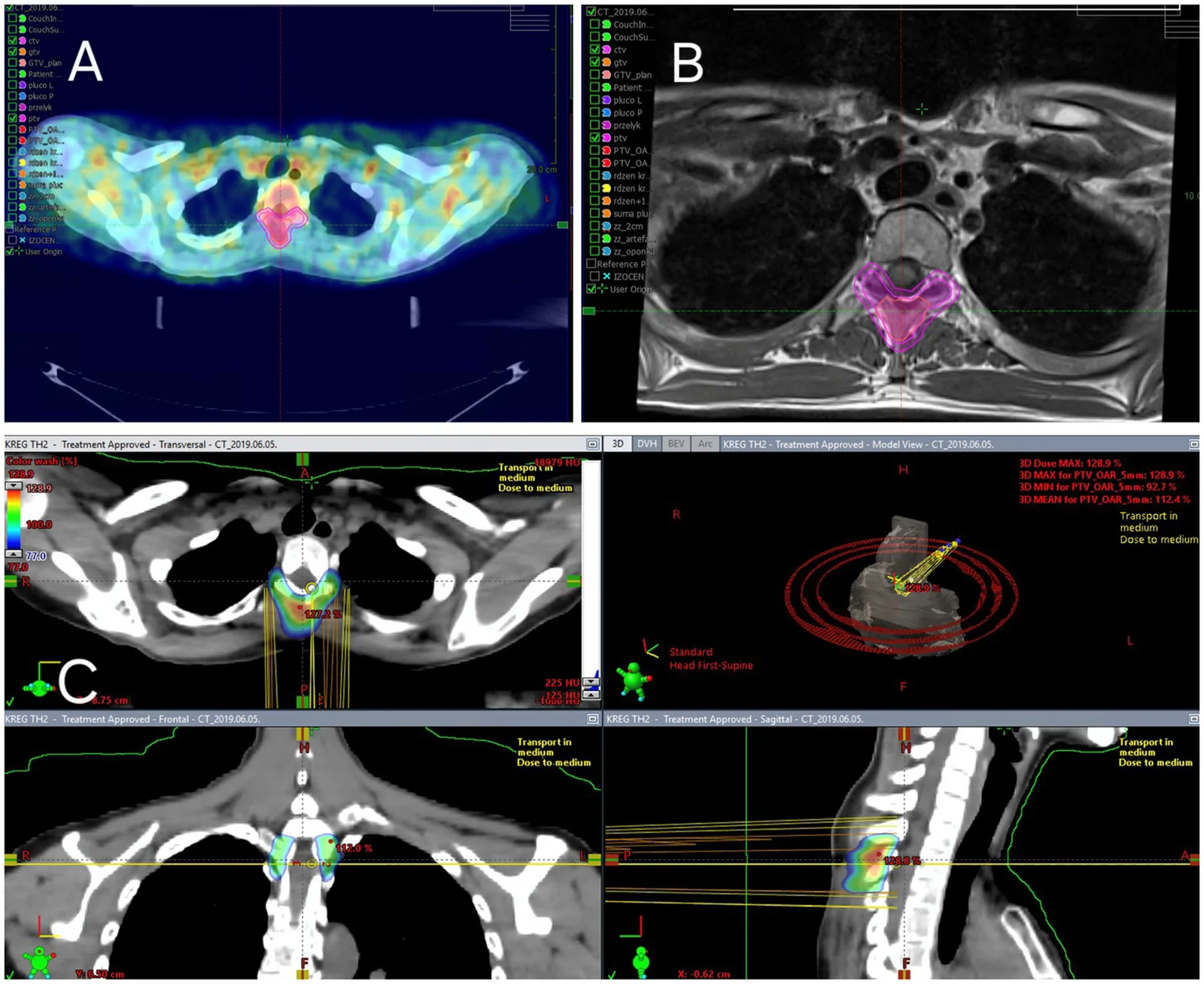
First spine SBRT (July 2019). **(A–C)** Target delineation at the T2 post-resection bed on CT-PET and CT-MRI fusion, showing gross tumor volume, clinical target volume, and planning target volume.

Subsequent monitoring of the patient with a series of imaging studies revealed systemic progression with involvement of the liver and lymph nodes in late December of 2019. In February 2020, the patient initiated first-line palliative chemotherapy based on the GD regimen (gemcitabine 1000 mg/m², docetaxel 60 mg/m²). Treatment continued until July 2020, when, given the patient’s overall stabilization and reluctance to continue treatment, the decision was made to resume imaging surveillance. An MRI scan from April 2021 revealed reduced signal intensity on T1 and significant post-contrast enhancement within the arches of the Th2 vertebra, the left arch of the Th3 vertebra, and the transverse process. Additionally, an 11x11 mm focal area of post-contrast enhancement was observed in the surgical bed. The nature of the lesions remained ambiguous, precluding differentiation between recurrence and a reaction following RT. It was not until August 2021 that a PET-CT scan provided unequivocal confirmation of morphological and metabolic progression in the thoracic spine. At that time, the patient refused to continue chemotherapy and instead elected to undergo surgical intervention at her local medical facility. The surgical procedure to excise the Th2/3 tumor, accompanied by laminectomy and posterior transpedicular stabilization of the Th1-Th5 vertebrae was performed in October 2021. Subsequently, in November, due to impaired healing, the patient underwent revision surgery and a posterior Th1-Th5 respondylodesis with resection of another recurrence. In December 2021, the patient exhibited rapid progression with multiple in-transit lesions in the thoracic area (Th1-Th5), therefore, she underwent a procedure involving the removal of the multiple nodules and a myofascial-dermatoplasty in the thoracic spine. A histopathological examination of these samples revealed the presence of a malignant peripheral nerve sheath tumor. However, a histopathological consultation at our center confirmed the original diagnosis of high-grade osteosarcoma. Concurrently, from November to December, the patient underwent a radiotherapy consultation for to determine the feasibility of administering further RT to the thoracic spine. However, due to the rapid progression of the disease, the RT planning was interrupted, and in January 2022, the patient resumed her previous line of chemotherapy with the GD regimen. Once again the planning for second SBRT for the spine was initiated. Using VMAT with CBCT image guidance and a 6 MV photon beam, with target volumes defined on CT-MRI fusion (**Figure 2**), 32.5/45 Gy was delivered in 10 fractions over two weeks to the 100%/80% isodose lines. The gross tumor volume (GTV) corresponded to residual thoracic-spine tumor, the clinical target volume (CTV) encompassed adjacent involved vertebrae and postoperative changes with a clinical margin, and the planning target volume (PTV) added a geometric margin appropriate to the location.

**Figure 2:**
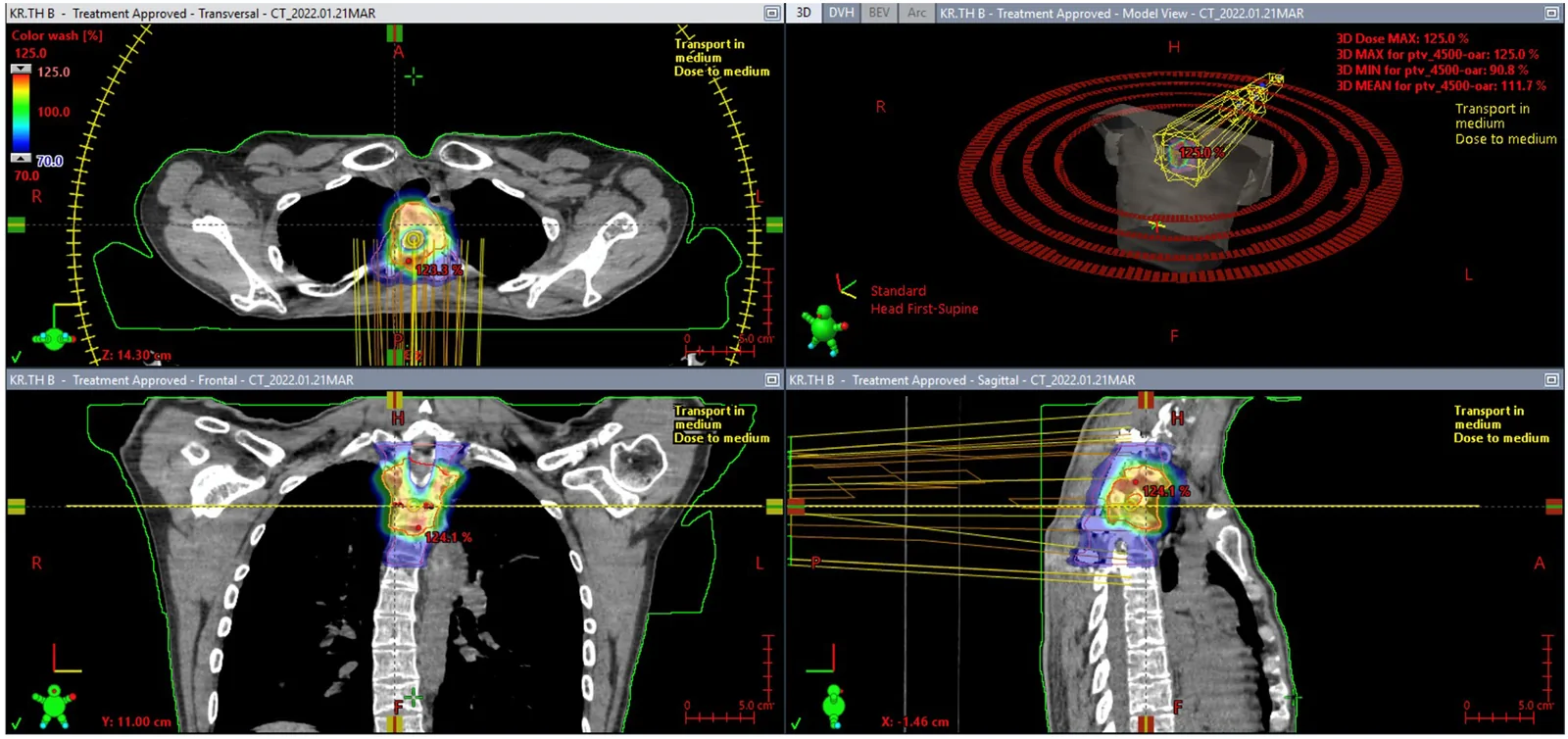
Second spine SBRT/reirradiation (January 2022). Target delineation on CT-MRI fusion for the thoracic spine.

Despite the SBRT, in March 2022, the patient exhibited persistent symptoms of spinal pain and lower limb weakness. A CT scan revealed PD with tumor spread to the lungs. Consequently, a decision was made to initiate second-line chemotherapy using the EI regimen (etoposide 100 mg/m², ifosfamide 1.5 g/m²). The patient received three courses of this treatment until July 2022. In July 2022, the patient was found to have further PD, as indicated by a CT scan. Consequently, the patient initiated a new line of chemotherapy, this time based on the AP3 (doxorubicin 20 mg/m² and cisplatin 30 mg/m²) regimen. In September, the patient’s treatment regimen was augmented with the addition of dacarbazine (200 mg), resulting in the patient receiving a total of 11 courses of chemotherapy. On November 2022, a CT scan revealed the presence of further PD in the lung lesions, an increase in the tumor infiltration within the Th2-Th4 vertebrae. Additionally, new metastatic lesions were identified in the cervical spine (C5-C7). Due to numerous previous surgical interventions and the technical impossibility of performing another surgery, the patient was qualified for another SBRT to the cervical and thoracic spine. In early December 2022, the patient underwent a third course of SBRT using VMAT with CBCT image guidance and a 6 MV photon beam, delivering 30/35 Gy in 5 fractions to the 100%/80% isodose lines (**Figure 3**); the GTV corresponded to tumor visible on MRI and the CTV added an approximately 1–2 cm margin, on the spine at the level of the C5-C7 vertebrae and the surrounding soft tissues. Spinal-cord and organ-at-risk constraints were respected, accounting for cumulative doses from prior courses. The cumulative biologically effective dose (BED), calculated using an α/β ratio of 2 Gy with Varian Velocity software, was 173 Gy for Dmax and 161 Gy for D_0.035_ cm³ for the spinal cord + 2-mm margin, while for the spinal cord alone, the corresponding cumulative BED values were 149 Gy for Dmax and 120.6 Gy for D_0.035_ cm³ (**Supplementary Figure 1**). In December, a new line of systemic treatment was initiated, employing paclitaxel (80 mg/m²) as the primary agent. The patient received a total of 11 treatment cycles until March 2023. Subsequent imaging revealed regression of the cervical spine infiltration and lung lesions, however the growth of two lesions in segment 3 of the right lung to 12 millimeters and in segment 9 of the left lung to 9 millimeters was diagnosed. Despite a favorable response to oncological treatment, later imaging revealed the loosening of a screw from spinal surgeries, with its protrusion extending out of the Th1 body on the left side, as well as pathological damage to the superior endplate of the Th3 vertebral body. These findings increased pressure on the nerves, resulting in a significant reduction in the patient’s quality of life, characterized by severe pain and paresis in the upper and lower limbs. Further diagnostic procedures revealed that the screw used to stabilize the upper thoracic spine had become loose, resulting in compression of the entire spinal cord. Consequently, in March 2023, the patient underwent surgical intervention involving posterior stabilization of C4-Th6 with Th3 osteotomy. Thereafter, and continuing until July 2023, the patient was maintained in a state of observation at our medical facility, with no additional therapeutic interventions administered, given the overall severity of her condition. The patient ultimately succumbed to the disease in February 2024.

**Figure 3:**
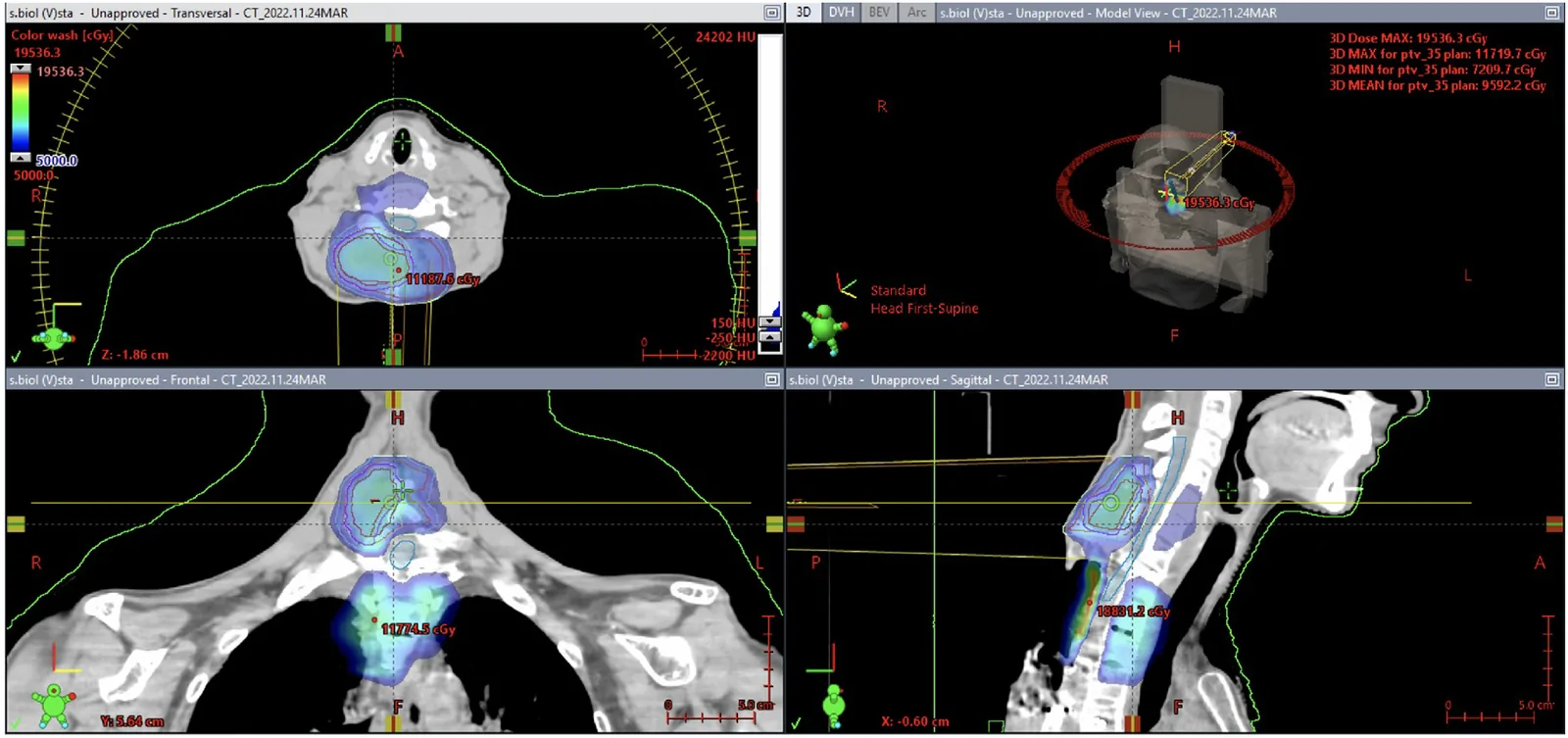
Third spine SBRT (December 2022). Target delineation for the lower cervical/upper thoracic spine.

An approximate timeline of the presented case is given in **Figure 4**, and technical characteristics of the SBRT plans is given in **Supplementary File 2**.

**Figure 4:**
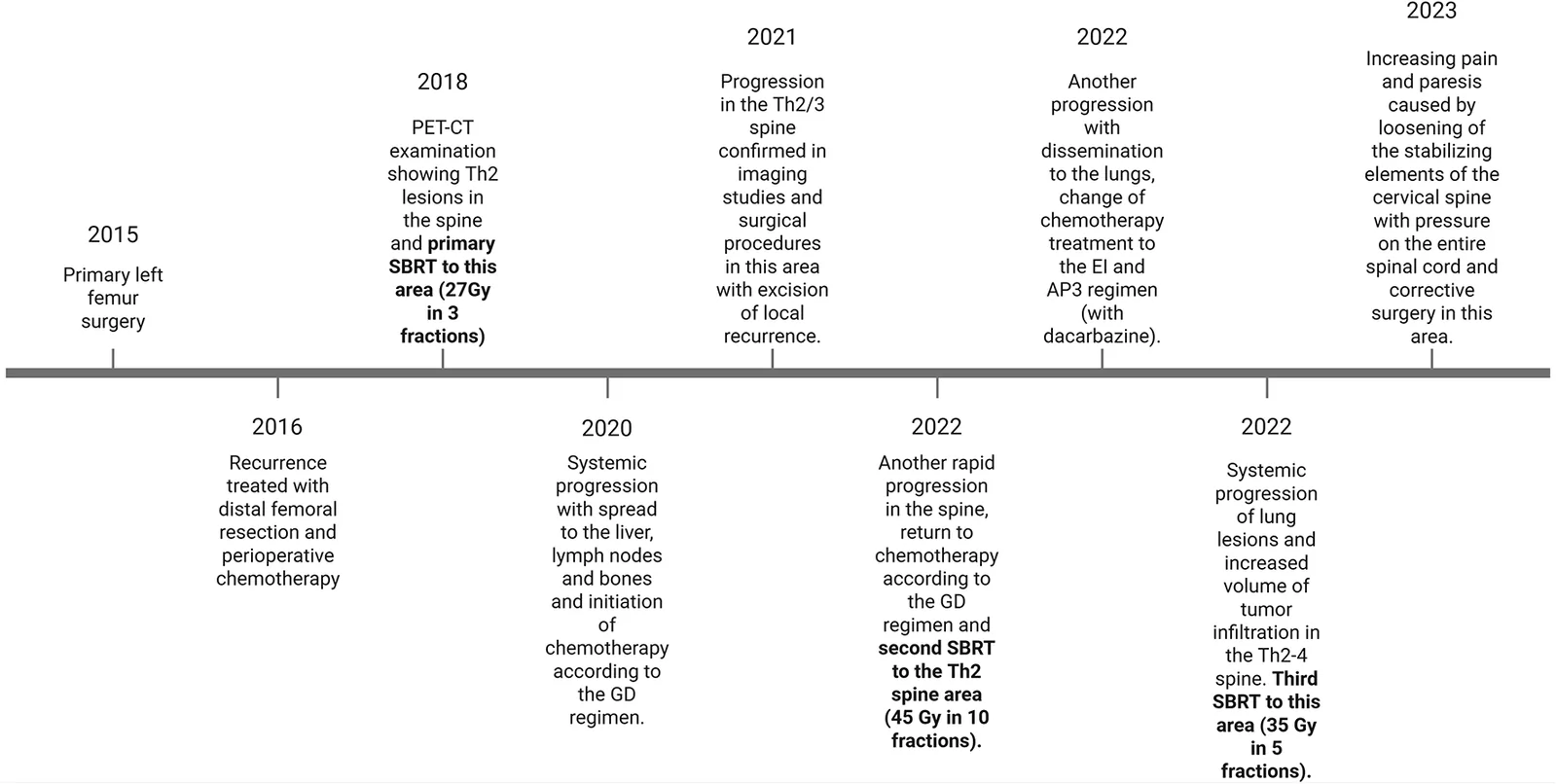
An approximate timeline of the presented case report, listing the most important events related to the use of multiple Stereotactic Body Radiation Therapy (SBRT) to the thoracic spine, includes AP3, doxorubicin, cisplatin; EI, etoposide, ifosfamide; GD, gemcitabine, paclitaxel; Gy, gray; PET-CT, Positron emission tomography–computed tomography.

## 3 Discussion

The clinical management of primary spinal irradiation and reirradiation remains a challenging issue for medical practitioners. The advent of novel methodologies, such as SBRT, facilitates the escalation of tumor doses while concomitantly constraining spinal cord doses to a lower and more tolerable level. In the context of reirradiation, this is of paramount importance in minimizing complications and ensuring treatment tolerance to critical structures such as the spinal cord (14, 15). In this case report, we present the first documented example of multiple reirradiations to the spine for disseminated osteosarcoma. While data pertaining to other diagnoses remains scarce, clinicians have access to some preliminary practical recommendations developed by two professional associations, the European Society for Radiotherapy & Oncology (ESTRO) and the International Stereotactic Radiosurgery Society (ISRS) (16).

To date, only a few large studies have been conducted on reirradiation after SBRT. Thibault et al. conducted a study that involved the analysis of a group of 40 patients with advanced cancers and spinal metastases after prior SBRT for reirradiation with a salvage second SBRT course to the same spine level. Moreover, approximately 40% of patients in the study were initially irradiated with EBRT prior to SBRT, thus undergoing a total of three cycles of RT. The study primarily included patients diagnosed with renal cell carcinoma, non-small cell lung cancer, and thyroid cancer, and in most cases, metastases were located in the thoracic spine. The median total dose and dose per fraction utilized in the study were 30 Gy in 4 fractions. With respect to local control, the results were encouraging, with cumulative incidences of local progression of 14.3%, 19.4%, and 28.5% at 6 months, 12 months, and 24 months, respectively. No substantial adverse events, including myelopathy, were observed (17). Another study evaluating spine reirradiation enrolled 102 patients, primarily those who had previously undergone thoracic spine irradiation. In this study, the findings were analogous, with the 6- and 12-month cumulative incidences of local failure recorded at 4.8% and 6%, respectively. Seven patients developed radiation-related neuropathy, and one patient developed myelopathy. Vertebral compression fractures were observed in 16.7% of the patients (18). A subsequent study, however, conducted on a comparatively limited sample of 17 patients, including four with sarcoma, yielded contradictory outcomes. In that study, the researchers employed reirradiation using SBRT doses of 30 and 35 Gy in five fractions. The median dose to 95% of the PTV was found to be 26.7 Gy. The local failure rates at 12 and 18 months were 0% and 8%, respectively. In that study, radiation-induced radiculopathy occurred in 4 (21%) patients. Of these patients, three experienced upper or lower limb paralysis, which, according to the authors, indicated a serious risk of complications from the treatment (19). A comparable case report was presented by Bentahila et al., who described a patient with advanced, metastatic breast cancer who, after receiving EBRT, underwent reirradiation with SBRT and, upon progression, a third course of SBRT to the same area. The patient received EBRT to the Th7–Th9 region at a dose of 30 Gy in 12 fractions, followed by SBRT at a dose of 18 Gy in 3 fractions at 80% isodose. Following further progression in this location, an additional SBRT was administered at a dose of 30 Gy in 5 fractions at 80% isodose. Following the third RT course, no acute complications were observed. After an eight-month period of follow-up, the patient exhibited a complete response in the thoracic spine and no significant post-treatment neurological complications (20).

In the case of our patient, three courses of SBRT were administered to the Th2 region of the thoracic spine due to repeated treatment failures. The patient, diagnosed with osteosarcoma, exhibited advanced disease, ultimately manifesting multiple metastases in various organs. However, she also exhibited significant clinical symptoms resulting from the spinal lesions, including pain and weakness, which had a profound impact on her quality of life. Consequently, the decision was made to pursue additional treatment courses. The patient received systemic treatment, which included various lines of chemotherapy and surgical interventions in the cervical and thoracic spine. The initial adjuvant SBRT in this patient’s case involved the delivery of 27 Gy in three fractions. The subsequent definitive SBRT was administered approximately 2.5 years after the initial treatment and involved 45 Gy in 10 fractions. However, due to the occurrence of another failure and the non-achievement of Dmax for the spinal cord at this level, a decision was made to administer an additional SBRT at a dose of 35 Gy in five fractions. This approach aligns with the findings of retrospective studies conducted for this specific indication. The feasibility of delivering multiple SBRT courses to the same spinal level is heavily reliant on the continuous, time-dependent recovery of neural tissue tolerance. Radiobiological models of spinal cord re-treatment demonstrate that prolonged intervals between courses—such as the 2.5-year gap in our patient—allow for significant tissue recovery, provided that the initial and cumulative biologically effective doses (BED) are meticulously calculated and adjusted for compounding risk factors like concurrent systemic therapies and surgical interventions (21). Three months after the completion of SBRT, imaging studies revealed a partial response of lesions in the lower cervical and upper thoracic spine, as well as a response of lesions in the lungs. Notwithstanding the efficacy of the therapeutic intervention, the patient exhibited an overall poor physical state, characterized by paresis and pain arising from the loosening of the screws employed for spinal stabilization. This sequence of events ultimately necessitated additional surgical intervention for further correction. However, it is challenging to ascertain whether the RT administered played a contributory role to this complication, particularly in light of the numerous prior surgical interventions in this area. Regrettably, the patient did not complete the dedicated quality of life assessment scales during treatment. However, given the advanced stage of the cancer process and the demanding medical procedures resulting in significant disability and caregiver dependence, a substantial reduction in quality of life should be inferred. Consequently, it is not feasible to directly evaluate the impact of the treatments on the cancer process and to compare their precise impact.

We therefore present another case report after Bentahila et al, the first in osteosarcoma, confirming the effectiveness and safety of SBRT reirradiation for spinal metastases. As previously stated, the present report pertains to a highly radioresistant tumor, however the issue of reirradiation in other diagnoses is becoming increasingly prevalent (22, 23). Notwithstanding the scarcity of literature and research in this domain, preliminary guidelines suggest that this type of treatment should be offered to a carefully selected group of patients. However, it is imperative to adhere to the principles of spinal cord sparing and the proposed protocols developed by experts in this field (24, 25). To minimize the risk of radiation-induced myelopathy during sequential reirradiations, decision-making can be supported by advanced radiobiological models that compute time-interval dependent recovery of the spinal cord (26). By employing computational tools that calculate the biologically effective dose (BED) and apply clinical conservative factors for patients burdened with prior surgical interventions or neurotoxic chemotherapy, clinicians can establish safer dose boundaries. Such quantitative approaches are essential when planning multiple SBRT courses, ensuring that cumulative spinal cord limits are strictly respected even in the absence of comprehensive clinical guidelines. In light of the potential, although uncommon, occurrence of adverse effects, decisions regarding reirradiation, particularly subsequent SBRT courses, should be made in a multidisciplinary panel and with the patient’s informed consent.

## 4 Conclusion

In this particular case, re-irradiation following prior vertical SBRT was found to be a safe and effective treatment modality. The present case report demonstrates the feasibility of maintaining an adequate dose delivered to the target while sparing the critical organ, the spinal cord, even with repeated SBRT. However, given the patient’s significant disease burden, the case study presented does not represent an optimal scenario. Consequently, the conduction of clinical trials is imperative to ascertain the role of re-irradiation in this particular indication with certainty.

## Data Availability

The original contributions presented in the study are included in the article/**Supplementary Material**. Further inquiries can be directed to the corresponding author.
