# Supplementary material for: Multidisciplinary approach with stereotactic reirradiation to oligoprogressive spinal metastases from dedifferentiated osteosarcoma: a case report
Source: Front Oncol. 2026 Oct 26;16:1929146. doi: 10.3389/fonc.2026.1929146 (PMC13745222; doi:10.3389/fonc.2026.1929146)
Supplement: Supplementary file 1 [file DataSheet1.docx]

|  | **First RT course** | **Second SBRT course** | **Third SBRT course** |
| --- | --- | --- | --- |
| **Dose fractionation** | 3x9Gy/80% | 10x4,5Gy/80%  10x3,25Gy/80% | 5x7Gy/80%  5x6Gy/80% |
| **Technique** | SBRT | SBRT | SBRT |
| **PTV volume [cm3]** | 24,7 | PTV45 101,8  PTV32,5 270 | PTV35 35,4  PTV25 84 |
| **PTV D95%** | 13,4 | PTV45 25,2  PTV32,5 28,3 | PTV35 33,9  PTV25 28,9 |
| **PTV D50%** | 28,9 | PTV45 49,6  PTV32,5 34,4 | PTV35 38,8  PTV25 34 |
| **OAR spinal cord Dmax EQD2** | 22,7 Gy | 32,4 Gy | 18,6 Gy |

**Supplementary Table 1.** Technical characteristics of plans and dose parameters for all radiation treatments. Gy — Gray; cm^3^ — cubic centimetre; PTV- planning target volume; D_max_ — maximum dose; EQD_2_ — equivalent dose in 2 Gy; OAR- organ at risk; SBRT- Stereotactic Body Radiation Therapy.

**Supplementary Figure 1.** Sagittal and axial views of the cumulative BED distribution in the cervical–thoracic spine. For the spinal cord + 2-mm margin, cumulative BED was 173 Gy for Dₘₐₓ and 161 Gy for D₀.₀₃₅ cm³; for the spinal cord alone, the corresponding values were 149 Gy and 120.6 Gy, respectively.
